# Amyloids assemble as part of recognizable structures during oogenesis in *Xenopus*

**DOI:** 10.1242/bio.017384

**Published:** 2016-05-23

**Authors:** Michael H. Hayes, Daniel L. Weeks

**Affiliations:** 1 Molecular and Cellular Biology Program, Carver College of Medicine, University of Iowa, Iowa City, IA 52242, USA; 2 Medical Scientist Training Program, Carver College of Medicine, University of Iowa, Iowa City, IA 52242, USA; 3 Department of Biochemistry, Carver College of Medicine, University of Iowa, Iowa City, IA 52242, USA; 4 Department of Pediatrics, Carver College of Medicine, University of Iowa, Iowa City, IA 52242, USA

**Keywords:** *Xenopus*, Amyloid, Germinal vesicle, Nuclear particles, Oogenesis

## Abstract

A hallmark of Alzheimer's, Huntington's and similar diseases is the assembly of proteins into amyloids rather than folding into their native state. There is an increasing appreciation that amyloids, under specific conditions, may be non-pathogenic. Here we show that amyloids form as a normal part of *Xenopus* oocyte development. Amyloids are detectable in the cytosol and the nucleus using an amyloid binding dye and antibodies that recognize amyloid structure. In the cytosol, yolk platelets are amyloid reactive, as are a number of yet to be characterized particles. In the nucleus, we find particles associated with transcription by RNA polymerase I, II and III and RNA processing contain amyloids. Nuclear amyloids remain intact for hours following isolation; however, RNase treatment rapidly disrupts nuclear amyloids.

## Abstract (precis)

**Summary:** Non-membrane-bound nuclear particles in *Xenopus* oocytes responsible for RNA transcription, modification and processing contain proteins assembled into amyloids as part of normal development.

## INTRODUCTION

Amyloids have historically been associated with pathologic conditions, including those in Alzheimer's and Huntington's diseases (Knowles et al., 2014); however, selective examples in yeast, snails, fruit flies and bacterial biofilms show that proteins in amyloid configurations can provide important functions (Newby and Lindquist, 2013). Hundreds of proteins are capable of forming the characteristic cross β-strand structure that defines amyloids. In amyloid configuration proteins gain enhanced resistance to denaturants and proteases, a potentially useful property for macromolecular assembly and storage. Importantly, amyloid assembly is reversible via the action of disaggregases that release proteins to refold into what would be considered their native state (Tompa, 2012). Switching between a protein's native and amyloid state has been shown to influence cellular phenotype (Chernoff et al., 1995; Halfmann et al., 2012; Rambaran and Serpell, 2008), and a growing number of studies have identified proteins that form (Maji et al., 2009; Newby and Lindquist, 2013; Si et al., 2010), or can form (Kato et al., 2012), amyloids. Visualizing the general distribution of amyloids in whole normal cells has been difficult; however we show here that *Xenopus* oocytes contain pools of amyloid that may include particles essential for nutrient storage, gene expression and RNA processing.

## RESULTS AND DISCUSSION

### *Xenopus* oocytes contain nuclear and cytoplasmic amyloids

**Fig. 1.**
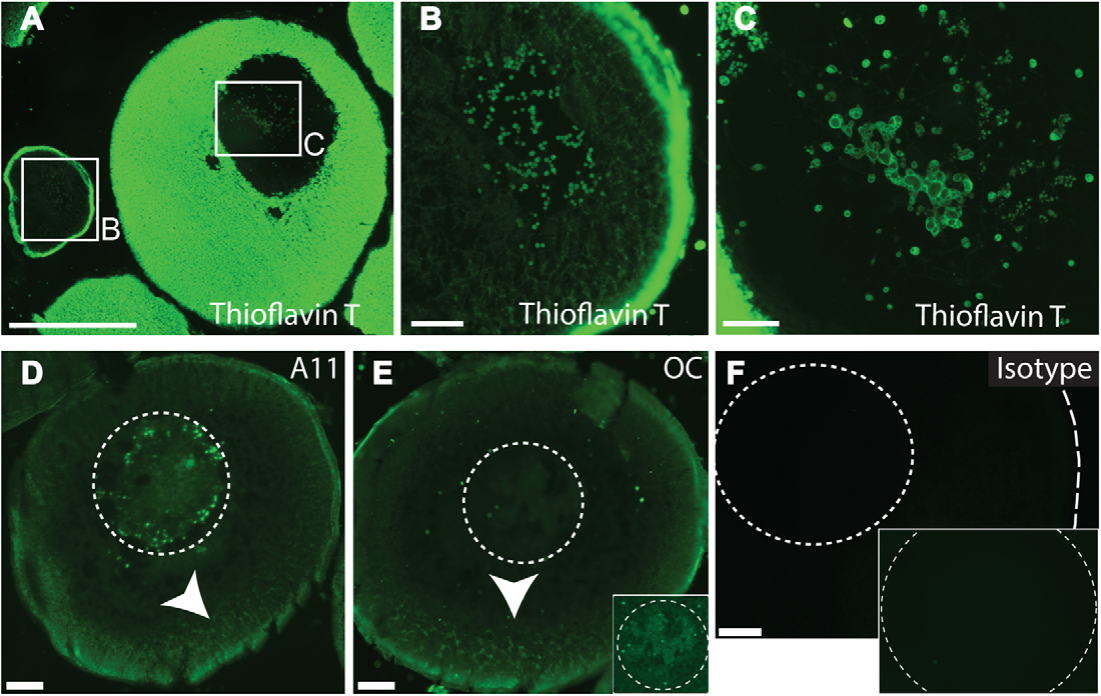
***Xenopus* oocytes contain nuclear and spatially localized cytosolic amyloid particles.** Sectioned ovary was probed for amyloids using thioflavin T (thio-T) or antibodies that recognize either oligomeric (A11) or fibrillar (OC) amyloid epitopes. (A-C) Thio-T staining in (A) highlights the difference in amyloid-positive yolk deposition as oocytes develop from stage II on (left) to stage VI (right). B and C show enlargements of the stage II (B) and stage VI (C) oocyte nuclear thio-T staining in A. Scale bars: (A) 500 µm and (B-C) 50 µm. (D,E) Antibody detection of amyloids in stage III oocytes using either an A11 (D) or OC (E) antibody reveals nuclear (dotted circles) and cytosolic staining of particles similar to that found in A-C, but with lower reactivity to yolk platelets. Arrowheads in D and E indicate increased staining in vegetal hemisphere of oocytes. (F) An isotype control antibody-stained sample. Insets in E and F depict increased exposure times to better visualize nuclear staining. Scale bars: 100 µm.

Thioflavin T (thio-T) selectively fluoresces when bound to the cross-ß strand structure of amyloid complexes allowing for differentiation of amyloid positive and negative structures (Groenning, 2010; Schmidt and Gorlich, 2015). Thio-T staining of cryofixed ovary sections reveals amyloid species in every stage of oocyte. Early pre-vitellogenic oocytes, before yolk deposition, show amyloid particles in both the cytosol and the nucleus (Fig. 1A left, magnified in B). Late stage VI oocytes demonstrate maintenance of nuclear amyloid throughout oogenesis (Fig. 1A right, magnified in C) and strong cytosolic thio-T staining resulting from yolk deposition (Fig. 1A). Previous studies of amphibian yolk platelets used circular dichroism and ultrastructural analysis to show that yolk proteins adopt extensive β-sheet structure within a yolk platelet's crystalline lattice/fibrillar network (Franzen et al., 1970; Karasaki, 1963), so it is not surprising that yolk platelets stain positively with amyloid stains.

Like thio-T, amyloid-specific antibodies recognize nuclear and cytosolic particles. To confirm our thio-T results, we probed oocyte sections with control, anti-oligomeric and anti-fibrillar amyloid antibodies (Kayed et al., 2007) (Fig. 1D-F). Amyloid formation is progressive, assembling as monomers, oligomers, and then fibrils. The A11 antibody preferentially binds oligomers while the OC antibody recognizes fibrillar species. It was noted that amyloid antibodies react less intensely with yolk platelets as compared to thio-T. We speculate that because thio-T is a small molecule [4-(3,6-dimethyl-1,3-benzothiazol-3-ium-2-yl)-N,N-dimethylaniline] it maypenetrate yolk more easily than a large antibody complex, although an alternative explanation would be that the viscosity of the yolk allows even unbound thio-T to fluoresce (Stsiapura et al., 2008). One consequence of the reduced reactivity of amyloid antibodies to yolk is the accentuated detection of cytosolic non-yolk amyloids. Non-yolk cytosolic amyloids appear spatially clustered (Fig. 1D-E). Cytosolic amyloids may include P-granules, stress-granules (Decker and Parker, 2012; Gallo et al., 2008) or may have a role in the stabilization and sequestration of maternal mRNA.

### Nuclear amyloids are associated with sites of RNA processing and RNA polymerase I, II and III transcription

**Fig. 2.**
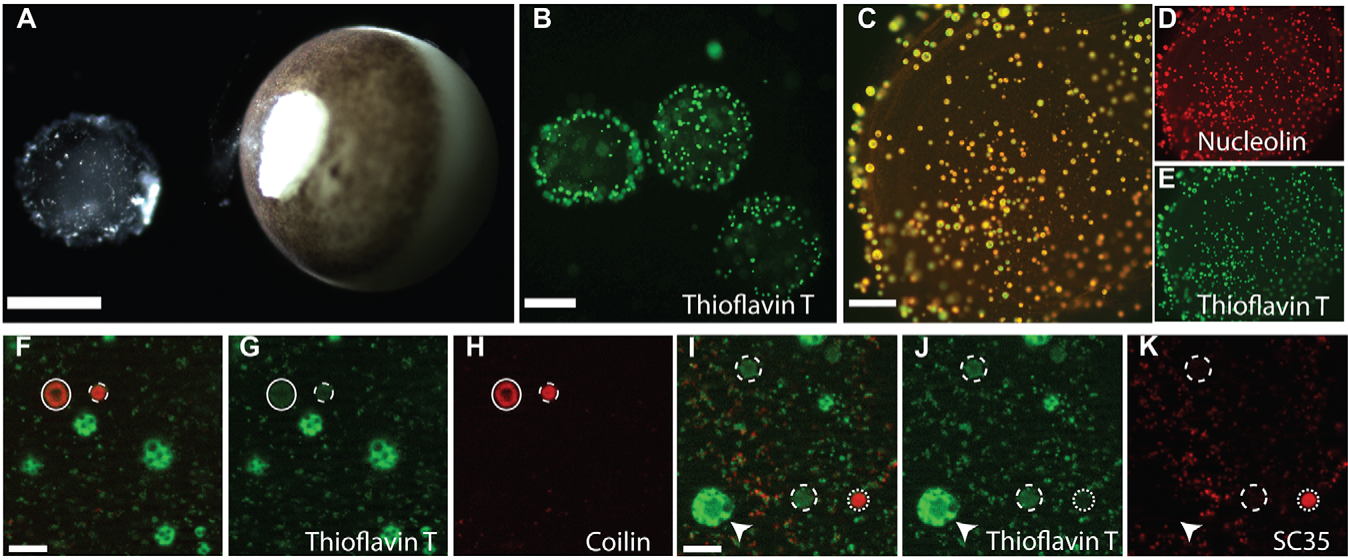
**Isolated *Xenopus* nuclei (GVs) can be used for combinatorial identification of nuclear particles with thioflavin T and particle specific antibodies.** (A) Manual removal of a GV from a stage VI oocyte. Scale bar: 500 µm. (B) Isolated GVs demonstrate amyloid containing particles seconds after thio-T staining. Scale bar: 200 µm. (C) Overlay of nucleolin immunofluorescence (red, D) and thio-T (green, E) staining of an isolated GV. Scale bar: 100 µm. (F) Overlay of images (G, green) stained with thio-T and (H, red) coilin immunofluorescence. A pearl particle is circled with a solid line, a histone locus body circled with a dashed line. Scale bar: 5 µm. (I) Overlay of panels (J, green) stained with thio-T and (K, red) SC35 immunofluorescence. Dashed lines indicate some thio-T-positive SC35-negative particles, dotted lines indicate a thio-T- and SC35-positive particle. The arrowhead points to a nucleolus in I-K. Scale bars: 5 µm.

Due to their large size, *Xenopus* oocyte nuclei, known as germinal vesicles (GVs), are an ideal system to study sub-nuclear organelles. To identify and characterize the amyloid content of nuclear structures we analyzed unfixed GVs (Fig. 2A).

When thio-T was added to GVs isolated from stage V or VI oocytes staining was essentially instantaneous (Movie 1). Both the pattern and number of amyloid-positive particles detected replicated the results seen with sectioned ovary (Fig. 2B, Fig. 1). GVs contain an actin-rich meshwork and numerous non-membrane bound multi-protein complexes associated with critical cellular processes (Feric and Brangwynne, 2013; Handwerger et al., 2005; Kiseleva et al., 2004; Mao et al., 2011; Mohamad and Boden, 2010). These complexes include: nucleoli, where RNA polymerase I transcribes and processes the RNA encoded by rRNA genes; nuclear speckles, that serve as centers for mRNA processing (Spector and Lamond, 2011); histone locus bodies, that are involved in the transcription and processing of histone gene mRNA by RNA polymerase II; and pearls, that coordinate RNA polymerase III transcription (Nizami and Gall, 2012). These particles can be identified by characteristic size, morphology, and the presence of signature proteins. These features, in combination with amyloid probes, allowed us to investigate which, if any, had amyloid.

Nucleoli, identified by nucleolin antibodies, demonstrate robust thio-T staining (Fig. 2C-E); however many thio-T-positive particles were not nucleolin-positive. To identify these particles we probed *ex vivo* GVs with an antibody that recognizes coilin, a protein present in histone locus bodies and nuclear pearls, and an antibody that detects SC35, a protein characteristically found in nuclear speckles (Nizami and Gall, 2012; Spector and Lamond, 2011).

Histone locus bodies (dashed line) and nuclear pearls (solid line) are thio-T-positive (Fig. 2F-H), while nuclear speckles (Fig. 2F-G, dotted line), stain less intensely. Histone locus bodies and nuclear pearls both contain the protein coilin; however histone locus bodies are homogeneously stained while only the periphery of nuclear pearls stain with coilin, thus creating a ring pattern (Nizami and Gall, 2012). The coilin-positive particles (red, Fig. 2H), are detected by thio-T (Fig. 2G, green), but are not as intensely stained as nucleoli (arrowheads).

*In vitro* experiments by others, using a chemical template or overexpression of an amyloidogenic peptide identified many proteins capable of forming or associating with amyloids. Among the proteins these studies identified were proteins present in nucleoli, histone locus bodies, pearls and nuclear speckles (Kato et al., 2012; Olzscha et al., 2011). Our data show that nucleoli, speckles, histone locus bodies and pearls are typically amyloid-positive in GVs. Fibrillar structure has been noted in nucleoli before (Franke et al., 1981; Monneron and Bernhard, 1969) and our findings indicate those fibers may be amyloid fibrils. We suggest that amyloid formation is beneficial and may help create a local environment aiding assembly, maintenance and function of nuclear particles.

### Nuclear particles have overlapping and distinct staining for oligomeric and fibrillar amyloid species

**Fig. 3.**
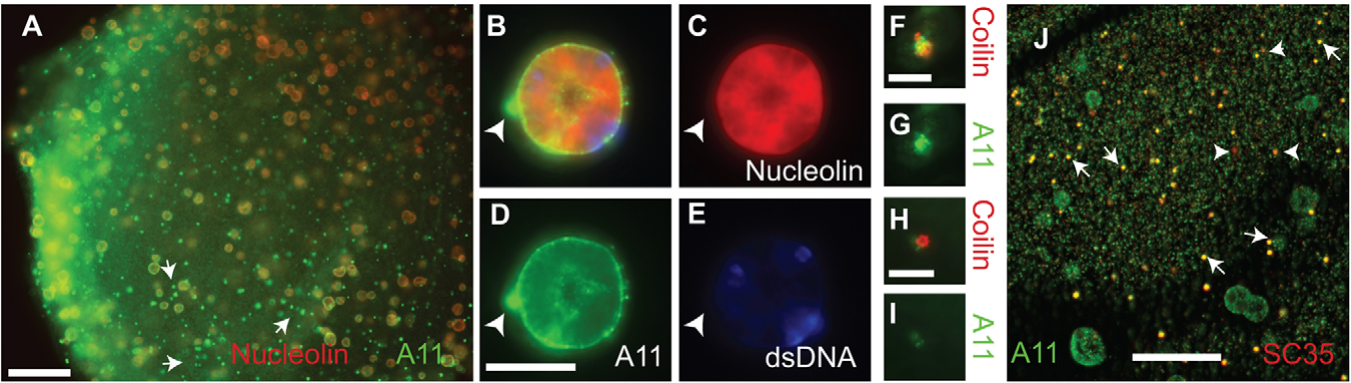
**Nuclear particles in isolated *Xenopus* nuclei (GVs) have overlapping but distinctive reactivity to amyloid detecting antibodies and thio-T.** Isolated GVs were examined using an anti-oligomeric amyloid antibody A11 and particle identifying antibodies. Panel (A) shows a low magnification view of a GV using A11 (green) and anti-nucleolin (red) antibodies. Arrows point to some of the many nucleolin negative, A11 positive particles. (B-E) are higher magnification images of a single nucleolus with (B) representing the composite of (C) anti-nucleolin (red), (D) A11 antibody (green) and (E) anti-dsDNA (blue) staining. The arrowhead points to an A11 positive sub structure. Scale bars (A) 500µm and (B-E) 10µm. Coilin positive particles, histone locus bodies (F-G) and pearls (H-I) are shown as overlays of coilin (red) and A11 (green) in (F and H). The A11 signal of the histone locus body (G) is higher than that of the pearl (I). Scale bars: 5µm. The composite image of A11 and anti-SC35 used to detect speckles in (J) highlights the variable intensity of the A11 signals. In (J) speckles with low A11 signal are indicated with arrowheads and those with more robust A11 signal with arrows. Scale bar: 50μm.

Nuclear particles were probed with anti-amyloid antibodies. Indirect immunofluorescence detection of nucleolin (red) and oligomeric amyloid (A11 antibody, green) confirmed that nucleolin positive particles contain oligomeric amyloid (Fig. 3A, overlay of treatments yellow/orange). A triple antibody-stained nucleolus (Fig. 3B) and isolated images provide a closer view of the arrangement of nucleolin (red, Fig. 3C), amyloid (green, Fig. 3D) and dsDNA (blue, Fig. 3E). A smaller amyloid positive but nucleolin and dsDNA negative particle was associated with the nucleolus (arrowhead, Fig. 3B-E), demonstrating the ability to differentiate sub-nuclear organelles with our protocol.

Coilin-positive structures differ in reactivity with the A11 oligomeric amyloid antibody. A histone locus body, identified by its homogeneous coilin staining, exhibits robust A11 staining (Fig. 3F,G). Pearls, with their distinctive ring shaped coilin staining, stain less intensely with A11 (Fig. 3H,I). Fig. 3J overlays anti-SC35 (red) and anti-amyloid antibody A11 (green), revealing yellow/orange speckles positive for both. When using this antibody combination, SC35-positive particles have variable A11 antibody reactivity (Fig. 3J).

Nucleoli, coilin-positive particles and nuclear speckles also react with the OC, fibrillar amyloid antibody (Fig. S1A-F), however the OC staining pattern is more punctate and variable. Additionally, we have observed coilin- and OC-positive puncta embedded within thio-T-positive nucleoli demonstrating distinct amyloid domains (arrowheads, Fig. S2A-F). This variable amyloid staining of nuclear particles suggests that there may be unappreciated diversity within these structures meriting further investigation.

### RNA is required for maintenance of nuclear amyloids

Nucleic acids have been implicated in the formation of nuclear particles and can induce proteins to form amyloids *in vitro* (Audas et al., 2012; Di Domizio et al., 2012b)*.* Nuclear particles contain a variety of nucleic acids, including extrachromosomal DNA, snRNA and rRNA. In particular, RNA has been implicated as critical component for the assembly of some proteins into amyloid structured hydrogels (Kato et al., 2012).

**Fig. 4.**
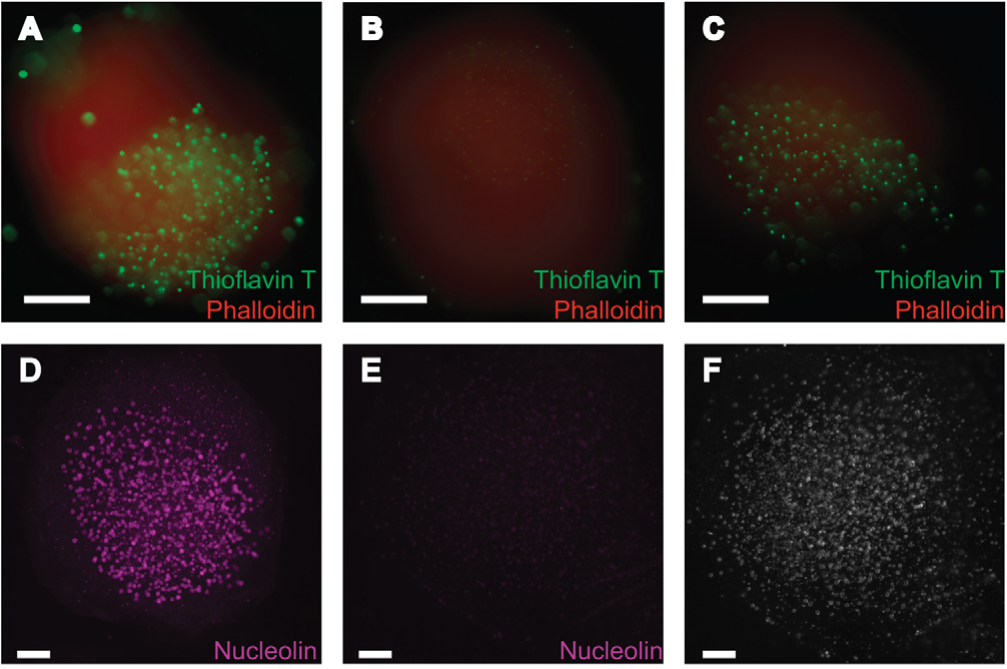
**Maintenance of nucleolin and thio-T staining of isolated *Xenopus* nuclei (GVs) is RNA dependent.** (A-C) Alexa Fluor 568 Phalloidin-stained (red) stage V-VI *Xenopus* GVs were left untreated (A) or treated with RNase A (B) or Xrn1 (C) for 30 min in the presence of 50 μM thio-T (green). Scale bars: 100 μm. (D-F) Untreated (D) or RNase A-treated (E) GVs were immunostained for nucleolin (magenta). (F) Dark field image nucleus. Scale bars: 100 μm.

RNase A (a single stranded endo-ribonuclease) treatment of GVs drastically decreased, but did not totally abolish, thio-T staining (compare Fig. 4A,B). The effectiveness of RNase A-mediated loss of RNA in nucleoli was confirmed by staining with SYBR Green II (Fig. S3). To determine if rRNAs or another species are responsible for this phenomenon we employed Xrn1 digestion.

Xrn1 is a 5′-3′ exoribonuclease requiring a terminal 5′ monophosphate, thus it will digest rRNA, excised introns and uncapped or cleaved mRNAs, but not capped mRNAs or 5′ trimethyl capped snRNAs. Xrn1 treatment reduced the thio-T staining intensity of GVs (Fig. 4C), but not to the level of that seen with RNase A. The effectiveness of Xrn1-mediated loss of RNA in nucleoli was also assayed by staining with SYBR Green II. In this case, we saw little overall change in SYBR Green II signal (Fig. S3). The retention of RNA in the presence of Xrn1 indicates that most probably, substrates like uncapped rRNA have protected 5′ends in nuclear particles. Interestingly, the Xrn1 treatment also indicates that partial disruption of amyloid content is possible.

Along with the loss of amyloid staining, nucleoli treated with RNase A lose nucleolin (Fig. 4E). Others have shown that nucleolin association with nucleoli is dynamic (Chen and Huang, 2001) and that fixed, isolated nucleoli when treated with RNase or DNase have structural changes (Franke et al., 1981). The GV isolation protocol we used leads to apparently stable association of nucleolin with nucleoli. That association is disrupted when RNA is lost. Interestingly, nucleolin was one of the proteins identified by Kato et al*.* that could be induced to form amyloid-like hydrogels as well form RNA:protein granules (Kato et al., 2012). Dark field microscopy of untreated and RNase A-treated GVs shows that RNase digestion decreases nucleolar amyloid content without disrupting the nucleolus entirely (Fig. 4F; Fig. S4), suggesting amyloid is not the only structural component of nucleoli.

Isolated GVs were also treated with DNase I to examine if the loss of DNA altered amyloid reactivity in nucleoli. These treatments were very effective in degrading chromosomal DNA, but neither completely depleted nucleoli of DNA, nor caused evident changes in thio-T staining. It is noteworthy that the DNase I treatment did qualitatively change DNA appearance in nucleoli, with a loss of clustered DNA positive areas even though general reactivity with DNA antibodies remained (Fig. S4). It is also noteworthy that DNA is not lost from nucleoli in GVs treated with RNase A, even though thio-T staining is greatly diminished (Fig. 4; Fig. S4). Taken together, these studies indicated that simply having DNA present within the nucleoli was not sufficient for retention of amyloid probe reactivity.

### Perspectives on these findings

Maternal amyloids persist into early embryonic development (M.H.H and D.L.W., unpublished), expanding their role beyond oogenesis. Amyloid-containing complexes may serve as a source of epigenetic information, be a method to package and protect nucleic acid (Di Domizio et al., 2012a,b; Zappulla and Cech, 2006) or act as a reservoir of inactive proteins as has been reported for some peptide hormones (Maji et al., 2009). Holding proteins in an amyloid state could regulate their distribution or the distribution of molecules they associate with, and allow for efficient and selective activation during the rapid expansion of cell number seen in early development.

Recent studies suggest that amyloid and disordered protein aggregates establish a size selective barrier function in nuclear pore complexes (Di Domizio et al., 2012a,b; Schmidt and Gorlich, 2015; Zappulla and Cech, 2006). One could imagine that amyloid assembly could control diffusion in nuclear particles facilitating an increase in local reactant concentrations while simultaneously conferring selective entry and exit of molecules to influence reaction rates and equilibria within particles.

Additionally, we note that there is a growing interest in the possible role of aggregates as a component of intercellular granules exhibiting liquid droplet characteristics (March et al., 2016; Weber and Brangwynne, 2012). Our data are consistent with the non-membrane bound nuclear particles often cited as having liquid droplet behavior also featuring amyloid content.

Our findings open a new avenue for the study of the assembly, disassembly and function of non-toxic amyloid formation using *Xenopus* as an experimental system.

## MATERIALS AND METHODS

### Oocyte isolation

Wild-type *Xenopus laevis* frogs were obtained from *Xenopus* 1 (Dexter, Michigan). Ovarian tissue was surgically removed and immediately placed into Oocyte Ringers solution (OR2, 82.5 mM NaCl, 2.5 mM KCl, 1 mM CaCl_2_, 1 mM MgCl_2_, 1 mM Na_2_HPO_4_, 5 mM HEPES and NaOH to pH 7.8). Ovarian lobes were dissected and incubated at 13°C for up to 5 days. All animal protocols were reviewed and approved by the Animal Care Office at the University of Iowa.

### Antibodies

The A11 anti-amyloid oligomer and OC anti-fibrillar amyloid antibodies (Millipore AB9234 and AB2286) were used at 1:500 dilutions. Hybridoma supernatants (DSHB, University of Iowa) for Nucleolin (B6-6e7 and P7-1A4) and dsDNA (autoanti-dsDNA) were used at a 1:5 dilution. The Coilin antibody (H1, Santa Cruz Biotechnology) was used at a 1:50 dilution, and the SC35 antibody (Pierce) was used at a 1:500 dilution. Anti-rabbit Alexa Fluor 568 and anti-mouse Alexa Fluor 488, 546 and 647 secondary antibodies (Molecular Probes) were diluted 1:500 in OR2. FITC and Cy5 conjugated anti-mouse secondary antibodies (Jackson Immuno Research Labs) were used at a 1:500 and 1:200 dilutions, respectively.

### Slide preparation and staining

A 0.5×0.5 cm piece of ovary was embedded in O.C.T (Tissue-Tek), frozen in liquid nitrogen, cryosectioned at 20 µm and dried overnight at 4°C. Sections were rehydrated in OR2, stained with 50 µM thio-T (Sigma) and imaged. For antibody staining, rehydrated sections were blocked in OR2 supplemented with 0.5% Triton X-100 and 1% goat serum, incubated in primary antibodies dissolved in blocking buffer, washed three times with 0.5% Triton X-100 supplemented OR2, incubated with appropriate secondary antibodies, washed three more times, and then imaged.

### *Ex vivo* thio-T assay

Germinal vesicles (GVs, nuclei) were manually isolated from stage V-VI oocytes and incubated in OR2 medium to allow intra-nuclear actin to polymerize. Nuclei were then placed into a depression slide with 50 µM thio-T and immediately imaged.

### *Ex vivo* immunofluorescence

Isolated GVs were placed in depression slides containing primary antibody for 15-30 min at room temperature, washed at least twice with OR2, and then probed with a fluorescently labeled secondary antibody. GVs were thoroughly washed in fresh OR2, mounted in Fluoromount G (Electron Microscopy Sciences), and gently coverslipped. Two rounds of primary and secondary antibody staining were performed in experiments using three primary antibodies. For experiments requiring antibody and thio-T co-staining the mounting medium was supplemented with thio-T to a final concentration of 50 µM. GVs can be stained with an intact nuclear envelope. Nuclear envelopes were removed prior to imaging.

### Nuclease treatment and staining

Isolated GVs were placed into OR2 with Alexa Fluor 568-conjugated phalloidin (Molecular Probes). Stained nuclei were washed in OR2 then placed into depression slides containing thio-T supplemented OR2, OR2 with 1 ng per μl RNase A (Sigma) or 1 ng per μl DNase I (Worthington Biochemical Corp) or New England Biolabs (NEB) buffer 3 with 2 units Xrn1 (NEB). Samples were incubated at 37° for 30 min then imaged. In parallel, nuclease-treated GVs were assayed for the effects of nuclease treatment via incubation in OR2 supplemented with 1× SYBR Green II (Molecular Probes) for RNA or immunofluorescent staining for dsDNA, as described above.

### Image acquisition and processing

Fluorescent images were acquired with AxioPlan or ApoTome fluorescent microscopes using an AxioCam MRm or AxioCam MrC 5 camera and AxioVision software (Zeiss). Images were processed and pseudocolored using ImageJ (National Institutes of Health) or Photoshop (Adobe Systems Inc).
